# Thai men’s experiences of alcohol addiction and treatment

**DOI:** 10.3402/gha.v7.23712

**Published:** 2014-05-19

**Authors:** Kulnaree Hanpatchaiyakul, Henrik Eriksson, Jureerat Kijsompon, Gunnel Östlund

**Affiliations:** 1 School of Health Care and Social Welfare, Mälardalen University, Eskilstuna, Sweden; 2 Basic Concept of Nursing Practice Department, Boromarajonani College of Nursing Changwat Nonthaburi, Nonthaburi, Thailand; 3 Swedish Red Cross University College, Stockholm, Sweden; 4 Praboromarajchanok Institute for Health Workforce Development (PIHWD), Ministry of Public Health, Nonthaburi, Thailand

**Keywords:** alcohol addiction, homo-social, hegemonic masculinity, alcohol treatment, barriers

## Abstract

**Background:**

Men are overrepresented with regard to alcohol addiction and in terms of alcohol treatment worldwide. In Thailand, alcohol consumption continues to rise, but few of those afflicted with alcohol addiction attend alcohol treatment programs, even though there is universal care for all. No comprehensive studies have been done on men’s experiences with addiction and alcohol treatment programs in Thailand.

**Objective:**

The aim of this study was to explore men’s experiences in terms of the ‘pros and cons of alcohol consumption’ in order to identify the barriers that exist for Thai men with regard to alcohol addiction and the decision to stop drinking.

**Design:**

Purposive sampling was applied in the process of recruiting participants at an alcohol clinic in a hospital in Thailand. Thirteen men with alcohol addiction (aged 32–49 years) were willing to participate and were interviewed in thematic interviews. The analysis of the data was done with descriptive phenomenology.

**Results:**

Through men’s descriptions, three clusters of experiences were found that were ‘mending the body’, ‘drinking as payoff and doping related to work’, and ‘alcohol becoming a best friend’ as ways of describing the development of addiction.

**Conclusions:**

The results highlight the importance of addressing concepts of masculinity and related hegemonic ideas in order to decrease the influence of the barriers that exist for Thai men with alcohol addiction with regard to entering treatment and to stop drinking.

Globally, excessive alcohol consumption has had a major impact on the lives of individuals, their families and communities, and on their physical and mental health. It is a causal factor in more than 60 major types of diseases and injuries (1). Men have a five-time higher prevalence for alcohol consumption and related problems than women; however, this trend is changing, with an increase in drinking by more young women in recent years in Thailand (2–4). The most common problems were identical for both men and women drinkers, namely the effect of drinking on work and finances, a lack of study or employment opportunities leading to increased drinking, and feeling guilt or remorse about drinking. For adolescents, males and females experienced different problems: fighting was the most frequent problem among males while drinking, and females suffered feelings of guilt or experienced a sense of remorse about drinking alcohol (2, 4).

Currently, in Thai society the consumption of alcohol is a widely accepted social activity. Thai people use alcohol as a mode of recreation and to enhance interactions among people in the community, specifically on holidays and at parties (4). In general, there is a forgiving attitude about social drinking with regard to men, though a more socially strict attitude exists toward women who drink. Research has found that women mostly drink at home and at parties, while men often drink at the workplace and in bars (4, 5). Moulinsart and Jongudomkarn et al. (6, 7) explained that the extent of a man’s daily consumption was an ingrained part of northeastern culture in Thailand and was related to various functions. For instance, these people believe that alcohol can be used for medicinal purposes such as a sleep inducer, appetite enhancement beverage, muscle relaxer, or for improved blood circulation. It is also commonly consumed as a refreshment to quench thirst and to accompany meals, particularly in the evening.

Although Buddhism, the main religion in Thailand, requires that good persons abstain from intoxicating their minds, drinking is overt and easily accessible (8, 9). Thamarangsi (8) pointed out that Chinese drinking culture has influenced Thai drinking patterns ever since the Ayuthaya period (1350–1767). At that time, Chinese migrants established the alcohol market and introduced the distillation technique for manufacturing spirits. Since then the alcohol market has been growing and has a huge impact on Thai society (8), such that drinking alcohol is now part of the cultural traditions in both China and Thailand. Obviously, Chinese traditional medicine has made use of alcohol as a major solvent in herbal medicine preparations (10).

Thailand has provided universal health care since 2002. As a result, people have had access to health care services through registered government facilities or non-government facilities with very little cost to themselves (11). Although the universal coverage scheme includes alcohol and substance treatment, only a small number of alcohol dependent patients, most of whom are men, actually receive specialty treatment for alcohol abuse (12). It seems as though the majority of the treatment for alcohol addiction is focused on physical disease rather than addiction, which requires long-term and continuing care (1).

## Men’s alcohol consumption

Health beliefs and behaviors among Thai men are heavily influenced by culture, as are the consumption of alcohol and patterns of seeking treatment for alcohol dependence. Men use general practitioner care service less frequently than women, and when they do seek health care services they are more likely than woman to focus on physical problems and less likely to discuss mental or emotional problems (13). ‘Authentic’ masculinity is constructed in part through the sharing of drinking stories within homo-social groups, in which the body’s ability to tolerate alcohol is seen as unlimited (14, 15). Drinking ‘too little’, or not at all, is also connected to subordinate constructions of masculinity, suggesting ‘weakness’ (16–19).

Gender differences in alcohol consumption are universal. More frequent and heavy drinking occurs among men, while more long-term abstention occurs among women; no cultural differences or historical changes have entirely erased these differences (20). According to De Visser and Smith (21), alcohol consumption is seen to be a specifically masculine behavior, and men may use alcohol consumption to demonstrate a specific pattern of masculine competence. Homo-social interaction can therefore explain how alcohol consumption connects to the constructions of the male identity. Emslie et al. (17) have shown that middle-aged men believe that the shared consumption of alcohol is an integral part of creating and maintaining male friendships. Drinking with friends was constructed, and indeed justified, as a way of helping men talk to each other, and as a way of providing social support and improving moods.

## Barriers to accessing treatment for alcohol dependence

There seems to be negative public stigma and attitudes toward alcohol addiction, labeling those who are addicted as dangerous, unpredictable and untrustworthy (22). Certain impacts of this public stigma include the fear of being labeled as an addicted person, embarrassment, and the worry of being looked down upon by others (23). The individuals who are at risk of excessive drinking believe that the general public reacts negatively to any individual who seeks help for alcohol treatment within the primary and specialty care sectors (24). On the contrary, Zakrzewski and Hector (25) demonstrated that men’s justification for alcohol consumption involved the desire to find a nice feeling, at least temporarily, as drinking alcohol was thought to be fun and to help them to cope with their lack of self-esteem. Research has found that stigmatizing behavior by health care providers has a potential to increase patient frustration and to increase the risk of patients leaving treatment impulsively, acting inappropriately, or refusing to return to particular alcohol treatment settings (26). Studies of patients with alcohol dependence indicate that the stigmatizing behavior they experience when entering the detoxification unit negatively influences the person’s self-esteem and self-efficacy (27).

Barriers to alcohol treatment, as seen from the patients’ perspective, included concerns of privacy and the belief that treatment was either unnecessary or not beneficial, as well as other practical and economic impediments to participation (28). The most frequent claim by participants was that they ‘wanted to handle the problem on my own’ (25, 29–31). In addition to the issue of privacy concerns, time difficulties and a fear of treatment were also reported by several studies (30–32).

It is difficult to raise public awareness regarding alcohol-related health-risk issues, as alcohol consumption among men in Thailand is connected to public acceptance and cultural involvement. In order to provide suitable and effective treatment for alcohol addiction, it is necessary to have extensive knowledge of the pros and cons of drinking, the accessibility of alcohol treatment and the social context in which this alcohol use is grounded. The authors of this study have experience in treating alcohol addiction in both Thailand and Sweden, with relevant backgrounds in health research from the fields of medical anthropology, nursing and social work. Few studies of alcohol addiction have been conducted in Thailand, and it is important to uncover why many resist accessing available treatments. This study aimed to investigate the barriers to alcohol treatment, with regard to the ‘pros and cons of drinking’, in order to identify the relevant barriers that exist before, during and after alcohol treatment. A bottom-up approach was used in order to explore this topic through Thai men’s lived experiences.

## Method

The present study was conducted at a hospital in Thailand that treated alcohol addiction. Data were collected from December 2012 to January 2013. After receiving a formal letter from the researcher responsible for collecting the data, the director of the hospital granted permission for the study to be conducted. The head nurse assisted the researcher with selecting the specific patients who could describe their life experiences and provide rich data. The participants obtained both oral and written information on the aim and procedure of the study from the principal researcher.

### The sample

Purposive sampling was used with the following inclusion criteria: taking part in alcohol treatment, diagnosed as having an alcohol abuse and dependence disorder, and over 20 years of age. Those diagnosed with psychotic disorders were excluded from the study. During the 2 months of data collection, 18 potential participants were asked to participate in the study, 14 of which agreed. Of these 14 individuals interviewed, one was a woman who we later decided to exclude. Thus, 13 men with alcohol dependence took part in the study, aged 32–49 years. Seven men were single, four were divorced, and the remaining two were married (Table 1). All participants gave informed consent.

**Table 1:** Number of male interviewees undergoing alcohol treatment in Thailand (2012–2013)

No.	Age	Number being treated at hospital	Program of treatment	Status
1	40	2	Detoxification	Married
2	34	Over 30	Detoxification	Single
3	34	3	Rehabilitation	Single
4	41	3	Detoxification	Divorce
5	36	3	Detoxification	Single
6	38	3	Detoxification	Single
7	39	1	Detoxification	Single
8	49	3	Rehabilitation	Divorce
9	34	3	Rehabilitation	Divorce
10	34	1	Detoxification	Single
11	43	3	Detoxification	Married
12	36	8	Detoxification	Divorce
13	32	6	Detoxification	Single

### The interviews

The interviews were conducted in a quiet room at the alcohol clinic. The researcher made contact with the participants by using ‘small talk’ and by asking permission from the patients to record the conversation. The questions asked were ‘Could you tell me about your experiences with regard to drinking alcohol and alcohol treatment?’ The interviewer (KH) kept an open-mind, listened carefully and encouraged them to reflect on their experiences more concretely by asking questions like ‘How do you feel?’ or ‘Could you please give me an example?’ Five participants were interviewed twice in order to get sufficient and rich data from each person; the other eight men were interviewed only once. Ten men had been admitted to the Detoxification Unit and three men had been admitted at the Rehabilitation Unit at the time the interviews took place. Each interview lasted at least 1 hour and 30 min. The 18 interviews were transcribed verbatim, resulting in a transcript of 203 pages.

### Data analysis

Phenomenological descriptive methodology was used to analyze the data. The method aims to find subjective experiences through established methods of bracketing, intuiting, analyzing, and describing. Such a philosophical framework is used to create meaning from actual lived experiences as revealed by the participants (33–35).

The interview transcripts were read several times to gain a sense of all of the material before starting the analysis. The first author listened to the tapes while reading the transcription and noted the various aspects captured during the interviews (34–36). During the reading of the transcripts, the overall meaning was identified through the differences and similarities recognized among the utterances. The text was then divided into small parts, so called ‘meaning units’. The meaning units were then classified into different subcategories. In this part of the analysis, the first author analyzed all the interviews as a whole, but there was continual movement from the whole to the parts and movement back to the whole. The data were kept at a concrete level, as far as possible, in order to minimize the effects of pre-understanding on the analysis. All meaning units were then translated into English and discussed with the co-authors. We reflected on all the meaning units by asking the specific question ‘What is this man really telling us?’ During these repeated discussions, subcategories and main categories began to emerge. As the analysis moved from a concrete to an abstract level of understanding, the essence was categorized in relation to each interview and in relation to the meaning units and categories (34–37).

### Ethical consideration

The study was approved in Thailand by Thanyarak Hospital Ethical Committee and in Sweden by the Uppsala Ethical Vetting Board, number 2012/493.

## Results

Thirteen Thai men described the pros and cons of drinking during hospitalization. They suggested that drinking is normative for men and that drinking was necessary in working culture in order to engage with colleagues and during occasions of celebration. They also described specific benefits from alcohol consumption, including relieving pain, reducing stress, and achieving balance their bodies that lead to increase work output. The cons of drinking described including being labeled by others, a loss of control, and being forced into treatment. Moreover, they also claimed that they received little benefit from the treatment.

By entering treatment in order to solve physical problems indicates that the patients were more interested in getting relief from their withdrawal symptoms than anything else. The treatment process had little influence on them, as they still believed that they could handle their drinking and that it was possible to return to drinking after treatment. This was also based on another important aspect of the men’s lived experiences of alcohol addiction, which included trying to keep alcohol as a part of their lives. Relapses and excessive consumption occurred when the men used alcohol during social activities or at work.

Three categories emerged in the men’s descriptions of the obstacles experienced before, during and after alcohol treatment: alcohol treatment as a ‘tool for mending the body’, alcohol as a method for ‘payoff and doping’, and alcohol as a best friend. These categories are described in detail below.

### Alcohol treatment as a ‘tool for mending the body’

Most of the men reported negative associations from being forced to attend various treatments by family members due to crisis situations. Although they recognized that heavy drinking was harmful for them, they often wanted to get through treatment in order to obtain control over drinking, not in order to stop drinking. Earlier experiences of treatment in the temple clinic provided by Thai traditional methods included the use of herbs, meditation, prayer, and spiritual Buddhist practices. One man claimed that he was concerned about having falsely promising the monks that he would stay sober; ‘If you wrongly swore it meant that you have sinned; I returned to drinking and never turn back to the temple anymore’ (Rew). Some men shared stories of friends that had returned to drinking after making such false promises and had died as a result of their drinking; these men never again swore to stop drinking, as they were worried about committing sin. Others compared their way of drinking with their relatives or friends who drank even more, but still appeared to be healthy.

Although treatment was effective in coping with withdrawal symptoms and improving body relaxation, it seemed to have little influence on long-term drinking habits. Most participants believed that they could control their drinking; for instance, if they restricted drinking to after work, they could satisfy their family’s expectations:

I believed it (alcohol) is a part of my life. I tried hard to control it, to the extent that it bears the least effect upon me. Although, I am here (the alcohol ward) I know I can’t stop drinking, but need to relax my physical state. So, I should drink in the evening, to compromise. (Arun)

Many of the men had previously attended alcohol treatment programs in the health care departments of hospitals due to a loss of bodily control or experiencing physical disorders. One man compared the treatment unit to a kind of quarantine center rather than a treatment facility; he did not believe it to be curative. The negative descriptions of treatment at the hospital included argumentative statements, such as the program is not useful, there is a lack of privacy, the treatment stay is boring, the professionals lack competence, the method in the alcohol treatment program is wrong or not clarified enough, and that the process is difficult to understand.

The program used was only for a short term period, and it was above my ability to understand. For the program to be effective, one need to have an able mind and more time, it was certainly a waste of time for them to get my attention and interest. They will never understand that. (Chon)

During treatment, the patients were most concerned with mending their bodies and relieving withdrawal symptoms and they wanted medicine offered for these purposes. The patients’ main intentions were to achieve bodily control and to be cared for according to their own wishes.

### Alcohol as a method for ‘payoff and doping’

While working, men noted that drinking alcohol was permitted during working hours, since that helped them work a bit more, at least in the short term. The patients worked as agricultural laborers, employees at the factory, on-site laborers, gardeners, and landscapers. According to these men, consuming alcohol helped them to relieve their muscle pain, stop shaking and reduce stress such that they managed to work even more. Some of the men stated that when they worked, they continually drank approximately 50–100 ml, four times per day, as can be seen in the following example:

I have worked in the rubber plantation and drank homemade white whisky to get a good feeling and muscle relaxation. After purchasing alcohol in the evening, it was divided into four little bottles and drunk four times during the day, in the evening, before bedtime at 11.00 p.m., all before work and during work hours. (Chuchai)

These men thought that the consumption of alcohol was a reasonable act in connection to their work. Several men with low-socioeconomic work as farm laborers consumed alcohol during the day and in the evenings in order to create a pleasant atmosphere, for refreshment and complete their work. Men with higher socioeconomic work, such as employers and officers, explained that they drank to maintain their social network and to satisfy their own cravings. Some men reported having muscle pains or shaking and they drank alcohol to balance their body and mind, and in order to be able to continue working; it could therefore be said that alcohol was used as a form of self-medication. During work, it was an act of generosity from a boss or a customer to provide alcohol as a payoff for work done, as well as a method for doping to ensure that each man worked to his full capacity. Several men explained that they receive alcohol and food from customers when working in the cadastral survey, paddy-farming, planting, or in gardening. One man stated that being a boss entails being friendly to the laborers, providing alcohol to make them get more work done. In addition, the boss wanted to create a good relationship with the workers by drinking with them, as the following example shows:

For those people, look at me, drunk all the time. The fact is that I just drank with my workers, purchased 200 baht worth of alcohol, for the work force to raise more money. So, I have to pay for them. (Tee)

As can be seen, drinking was regarded a method for ‘payoff and doping’ utilized by men of higher socio-economic for engaging with their employees. The tradition of giving alcohol is particularly used to stimulate laborers to increase their level of work output, as has been described earlier in this text (e.g. doping them to work longer hours). Another reason for providing alcohol to the work force is to be labeled as a generous and kind boss.

### Alcohol as a best friend

During the interviews, the participants expressed shame and embarrassment at not being able to handle their drinking, and perceived it as a sign of worthlessness. They also stated that this lack of control over their drinking resulted in negative judgments by others, which left them feeling isolated. Most of the participants described feeling lonely, lacking trust or caring feeling toward others, and stated that drinking released them from certain negative feelings. They also stated that although drinking sometimes felt more like a job or a nuisance and was no longer fun, they needed alcohol to survive and relieve pain. Two men described their depression and suicidal thoughts, even attempted suicide during bouts of heavy drinking. Another participant expressed his hopelessness in stopping to drink and another felt guilty regarding his consumption behavior. They also described experiences of stigmatization, like feeling dishonored which further lowered their self-esteem and increased their drinking, as stated by one of the participants:

No close friends were trusted to tell my private matters to. I looked at the trees, the fish, talked with the invisible things and just imagined they could understand. I drank and began thinking about the mistakes that I have done with people over the last year. What were my mistakes and who were the people that gave back to me. Why have I done these things? Feeling disappointed by my unexpected unemployment, I began to lead a dog and cat life, drinking heavily when socializing … whenever I ran into a good friend. (Son)

According to this quote, the man labeled himself a dog or a cat, meaning that he felt worthless, as he was dependent on his parents, had no close friends, experienced a loss of family, felt dishonored, and was unemployed. Most men preferred to stay home and drink alone in order to avoid getting into trouble when drinking outside the home, though they also often interacted with others while drinking. Despite family support for all of the men interviewed in treatment, most had suffered multiple failures in life, including failed marriages, work and previous treatment attempts. These feelings of embarrassment and shame lowered their self-esteem. Drinking alone was a way to ease life when feeling different or marginalized. One man explained how he hid alcohol from others in order to protect his self-esteem:

I bought it and hid it inside my shirt, fastened by the belt. I don’t want to be seen drinking. I drink but can do my work, like other people do. I was often cited as a good example of a sober drinker, unlike those who drink otherwise. (Chuchai)

Drinking when unemployed is looked down upon by society, according to Thai men. For this reason, the participants prioritized maintaining their jobs while simultaneously continuing to drink.

## Discussion

The ‘pros and cons of drinking’, according to the experiences of 13 Thai men, were explored in order to identify the relevant barriers that exist before, during and after treatment for alcohol abuse. These pros and cons were segregated into three categories: alcohol as ‘payoff and doping method’ (mostly pros), alcohol treatment as a ‘tool for mending the body’ (both pros and cons) and ‘alcohol as a best friend’ (both pros and cons).

The general reasons for drinking alcohol included peer pressure, relaxation after work, and reducing stress (5–7, 32). The participants also emphasized the value of drinking in facilitating socializing and enhancing interpersonal relationships. Other functions of drinking in social and cultural occasions include celebration, hospitality, and reciprocity. Thus, drinking alcohol can serve more than one function on any given occasion (6, 7).

The advantages of drinking can be understood by the theory of homo-social grouping, where men normalize alcohol consumption as part of the construction of male identity. Thailand has been described as a society based on the concepts of a patronage-driven culture, which values preconceived notions of high masculinity that are somehow associated with alcohol consumption. Thai men who are active in society and employed outside of the home ‘have to drink’ in order to meet the skewed cultural values of obsessive masculinity as they relate to alcohol consumption (6). Furthermore, drinking is utilized as a mediation device in developing relationships among Thai men. Offering alcohol at work or after work is a social construction that, according to the present study, is considered a sign of generosity and respect; for example, participants argued that being a boss entails providing alcohol and drinks to his crew, as a way to respect and honor them as men (14, 15). Thus, the findings illustrate that men’s drinking is part of the notion of hegemonic masculinity, where men drink socially with men.

The participants described the disadvantages of drinking, including being forced into treatment and issues of self-stigmatization. Interestingly, a positive outcome of treatment was the relief of withdrawal symptoms; withdrawal symptoms have been found to be the major concern in the acute hospitalization of people with alcohol addiction (38). According to Jakobsson et al. (19), many men are forced to seek treatment and they expressed shame and embarrassment as a result of their inability to handle alcohol, perceiving this failure as a sign of weakness. However, having a comorbid affective disorder or other problems directly attributable to alcohol use increases the likelihood that such individuals will seek treatment (13).

Although the participants had made several failed attempts to stay sober, they still believed that they could control their drinking. Unlike another research study, previous treatment attempts did not seem to improve the patients’ knowledge (23). The interviewed men were rather ashamed of their earlier treatment attempts, particularly having made false promises of sobriety to monks, since Buddhist precepts dictate avoiding intoxication, the drinking of alcohol and the taking of drugs (8, 9). Embarrassment and shame further damaged the men’s already low self-esteem and they described suffering from the social stigma associated with alcohol addiction, as have been found in previous research (22, 23, 27). Shame and a lack of self-esteem could combine to become one major barrier to the treatment of alcohol addiction. Research has found that men use health care services and seek help less frequently than women and are more likely to refrain from disclosing mental or emotional problems (13).

The health care services need to develop a gender relational perspective of masculinities and health (13, 15). In relation to men suffering from alcohol addiction, this would include critical investigation of the division of labor and domestic work as well as identifying the influence of socioeconomic differences among groups. The present findings provide vital insight into how men construct the drinking culture through their stories and how its link to male bonding in homo-social grouping behavior seems to be a crucial barrier to alcohol treatment and to abstaining from drinking among certain groups of Thai men. Risk-taking plays a role in constructing masculine identities, and the sharing of drinking stories that include risk-taking is integral in creating and maintaining male friendships (13, 17, 18). The themes of men’s stories reflect the pressures of male culture in terms of being the breadwinner and continuing to work, as was pinpointed by Connell and Messerschmidt (14). Hegemonic visions of masculinity seem to be of vital importance in alcohol addiction and ‘knowledge of doing gender’ needs to be included when health care services consider appropriate treatment.

### Clinical implications

The research was conducted at a hospital in Thailand, which includes a detoxification and rehabilitation unit for alcohol treatment. One limitation of the study was the small sample; however, the participants were heterogeneous and representative of Thai men who typically enter alcohol treatment programs. The interviews also provided rich and in-depth descriptions of men’s experiences of alcohol addiction and treatment programs.

In listening carefully to the men’s descriptions of addiction and of their struggles in life, new knowledge of Thai men’s alcohol and working culture was found. One apparent barrier during treatment was that men spent the majority of their time focusing on physical problems and not the social or psychological aspects of their addiction. This raises questions with regard to the level of the patients’ engagement in the treatment. However, the hegemonic masculinity construction and the act of drinking alcohol as a part of homo-social grouping at work among groups of Thai men can be understood as a barrier before, during and after treatment that needs to be further studied. Moreover, the act of entering alcohol treatment programs among Thai men is related to self-stigma.

The barriers found on an individual level included focusing on the body instead of the addiction behavior, drinking alcohol during work, and self-stigmatization and shame in relation to the treatment or the treatment attempts. These barriers highlight the importance of addressing masculinity and hegemonic ideas in order to decrease the influence of the barriers before, during and after treatment for Thai men with alcohol addiction.
